# Involve Residents to Ensure Person-Centered Nursing Home Care During Crises Like the COVID-19 Outbreak

**DOI:** 10.1007/978-3-030-65355-2_21

**Published:** 2021-03-20

**Authors:** Katrien Luijkx, Meriam Janssen, Annerieke Stoop, Leonieke van Boekel, Marjolein Verbiest

**Affiliations:** 1 Tilburg University, Tilburg, The Netherlands; 2 Tilburg University, Tilburg, The Netherlands; 3 Tilburg University, Tilburg, The Netherlands; 4 Tilburg University, Tilburg, The Netherlands; Tranzo Scientific Center for Care and Wellbeing, Tilburg School of Social and Behavioral Sciences, Tilburg, The Netherlands

## Abstract

Nursing homes aim to provide person-centered care and recognize residents as unique individuals with their own histories, life goals, and preferences. The life expectancy of nursing home residents is rather limited. Nursing homes have been hit hard by COVID-19 because of an increased risk of death and a total nursing home lockdown from March 19 until the end of May 2020. Although social relationships are a basic human need and the fulfillment of social needs is essential for both physical and mental health, nursing home residents were no longer allowed to meet their loved ones. This decision was taken without involving residents and their loved ones and without considering the psychosocial impact of such measures for residents and their loved ones. When visitors were again allowed in the nursing homes, this was valued highly. To enable decent decision-making, we call both the government and nursing homes to involve residents and their families in decision-making. It is essential to know how residents weigh the risk of a COVID-19 infection and the possible implication of them opposing social isolation. We have to adapt to a new common and need to stop talking about residents and their loved ones and start talking with them.

In the Netherlands, nursing homes provide residential and long-term complex care to older adults with impairments in different health domains. The life expectancy of nursing home residents is rather limited in general. For a few decades now, autonomy, well-being, and quality of life are increasingly being recognized as important in nursing home care, and the exclusive focus on enhancing safety, health, and longevity is no longer dominant. Although a medical perspective is still present in nursing homes because many residents need some form of medical care, the person-centered care (PCC) model is gradually being adopted in nursing home practice (Koren 2010). PCC aims to facilitate residents in living the life they desire and in being recognized as unique individuals with their own histories, life goals, and preferences (McCormack 2001). The various voices of residents need to be heard, and tailored care is essential but challenging for professional caregivers because of different individuals living in a group. Therefore, nursing homes struggle to bring PCC into practice although they embrace the idea.

## Nursing Home Lockdown

Nursing homes have been hit hard by COVID-19. Registration data show that, in the period from March 18 until May 13, 38% of the suspicions of COVID-19 infections in nursing homes were confirmed. The risk of death was three times higher for residents with confirmed COVID-19 infections compared to residents who tested negative. Male residents with a confirmed COVID-19 infection had a two times higher risk of death compared to female residents. Dementia, kidney failure, and Parkinson also increased the risk of death (Van Loon et al. 2020).

Worldwide restrictive measures, including social distancing, have been taken to protect public health and to flatten the curve. Medical insights, mathematical modeling, and opinions of the public and experts led the Dutch government to decide to close nursing homes on March 19 for everyone except professional caregivers providing essential care on a daily basis. From one day to the next, nursing home residents were no longer allowed to meet their loved ones and were sometimes even expected to spend their days in their own rooms or apartments. The high prevalence of poor physical health in nursing home residents dominated this decision without taking into consideration the psychosocial impact of such measures. It is still unknown to what extent the voices of individual residents or their loved ones have been taken into account in decisions with such a large impact.

## Social Relationships

Social relationships are a basic human need (Maslow et al. 1970). Unsatisfied social needs negatively impact both physical and mental health (e.g., Cacioppo et al. 2003) while satisfied social needs positively impact physical and mental health as well as well-being (e.g., Golden et al. 2009). Involvement of family members in nursing home care improves the well-being and quality of life of both residents and their loved ones (Janssen et al. 2011). Moreover, also for nursing home residents and their spouses, love, intimacy, and sexuality are fundamentally important aspects in their lives (Roelofs et al. 2017, 2019). To enhance person-centered nursing home care, it is essential to involve family members as well. It is important to involve both residents, and their family members to find out the preferences and needs of the residents. Therefore, nursing homes stimulate and facilitate family participation.

During the lockdown of nursing homes, staff and family members acknowledged the importance of social relationships. They searched for and found creative solutions for residents to talk to and be in contact with their loved ones while preventing physical contact. Examples are video calling facilitated by staff, welcoming visitors in specially designed spaces using Perspex to enable the eye to eye contact, and using a phone to listen to each other’s voices. Furthermore, aerial work platforms were placed to enable loved ones to see each other’s faces.

In contrast to community-dwelling older adults, nursing home residents and their loved ones did no longer have any autonomy in weighing the risk of a COVID-19 infection against the importance of fulfilling their social needs. They were unable to decide for themselves whether they wanted to meet loved ones or not and to touch, kiss, or hug each other or not. Individual residents might have preferred to meet with their loved ones and hug them, despite the risk of a COVID-19 infection. This meant that, even in the last phase of their lives, residents, and their loved ones including spouses with whom they had long histories had no choice nor voice in this matter.

## Visiting Arrangements

On May 11, a pilot started, covering 26 nursing homes spread over the country, to test whether it was possible to allow visitors in nursing homes without causing new COVID-19 outbreaks. Visiting arrangements varied among participating nursing homes but were all in line with the restrictive measures in place at the time. In the first weeks, only one dedicated visitor for each resident was allowed to visit his or her loved one. Visiting times varied among nursing homes from 30 to 60 minutes to no time restrictions at all. Only visits by appointment were possible due to a mandatory health check (Koopmans et al. 2020).

A general study covering all 26 nursing homes and an in-depth study including five nursing homes monitored the compliance to these restrictive measures and the impact of the visiting arrangements on staff, visitors, and residents by proxy (Hamers et al. 2020; Koopmans et al. 2020).

## Loved Ones Visiting Again

As expected, the study showed that visiting arrangements are highly valued by both loved ones and staff. Loved ones are very happy to visit their spouse or parent again. Especially when a resident has hearing problems and/or dementia, a visit in person is of much more value than video calling and all other creative solutions to meet social needs. Although the use of mouth masks or being unable to meet in the private room of the resident makes the visit somewhat impersonal, family members are relieved to meet their loved ones in person because they are aware of the short life expectancy of nursing home residents in general. During the lockdown, many family members worried whether they would ever be able to meet their loved ones in person again and whether they would still recognize them. For some family members the visit was confronting due to the visible (cognitive) health deterioration of the resident during the lockdown of the nursing home (Koopmans et al. 2020).

## Residents by Proxy

Loved ones and staff were asked about the impact on residents to meet their family after a long period. Due to ethical concerns in combination with the time pressure of the monitoring studies, it was impossible to observe or interview residents themselves (Hamers et al. 2020; Koopmans et al. 2020). Based on the insights of proxies, it is evident that the lockdown of nursing homes affected the well-being of residents negatively, increased loneliness and sometimes seemed to lead to a decline in health. Residents enjoyed being reunified with their families after a long and often lonely and uncertain time and were cheerful, livelier, and more active after having been visited. They are looking forward to the next visit. For some residents with dementia, visits were rather confusing and led to sadness and agitation because they did not really recognize their loved ones anymore, got overstimulated during the visit, and were constantly looking for their loved ones after the visit. For some of these residents it was, therefore, decided to reduce or even stop the visits (Koopmans et al. 2020).

## Involve Residents and Loved Ones

To enhance PCC, it is essential to know the needs, preferences, and possibilities of nursing home residents themselves, also in times of crises like the COVID-19 outbreak. Studies that compare the perspective of nursing home residents to that of proxies, for instance, loved ones or staff members, show that these perspectives differ (e.g., Dröes et al. 2006; Gerritsen et al. 2007). This nuanced difference may affect the resident’s experience of feeling heard, seen, and respected as a unique individual in the care and support he or she receives. Therefore, it is of utmost importance to study the perspectives of nursing home residents in general and on the closure of nursing homes in particular. Although it is ethically challenging and not easy to interview residents with dementia, it is possible (Roelofs et al. 2017). Nevertheless, the perspectives of nursing home residents during disasters or crises are scarcely studied (Van Boekel et al. 2020), which was also the case in the studies monitoring the Dutch visiting arrangements in nursing homes (Hamers et al. 2020; Koopmans et al. 2020).

Although nursing homes, supported by the national government, aim for adopting a person-centered approach, during the first peak of the COVID-19 outbreak, nursing home residents and their loved ones were not involved in decision making. That is a missed opportunity. We therefore call both the government and nursing homes to involve residents and their families in decision making both in general and in times of crises because it is essential to know how residents weigh the risk of a COVID-19 infection and the possible implication of them opposing social isolation. Against the background of their short life expectancy, they might prefer to meet loved ones despite the risk of a COVID-19 infection. A new outbreak of COVID-19 can be expected in the future and also within nursing homes, therefore we have to adapt to a new common. It is time to stop talking about residents and their loved ones and start talking with them. Involvement of the residents and their families in policies and daily care in the new common is necessary to maintain person-centered care in nursing homes.
